# Ground-based imagery dataset for early weed classification in tomato crops

**DOI:** 10.1016/j.dib.2025.112249

**Published:** 2025-11-12

**Authors:** Hugo Moreno, Gabriel Rivera, Dionisio Andújar

**Affiliations:** Centre for Automation and Robotics, Consejo Superior Investigaciones Científicas (CSIC), Ctra. de Campo Real km 0.200 La Poveda, 28500 Arganda del Rey (Madrid), Spain

**Keywords:** Summer row crops, Weeds identification, Deep learning, Neural networks, Object detection image

## Abstract

Accurate identification of weed species at early developmental stages is essential for advancing precision agriculture. Species-level classification enables site-specific management strategies, reducing herbicide use and promoting sustainable crop production. This study introduces a curated dataset of RGB images captured using a handheld Canon PowerShot SX540 HS camera, offering a spatial resolution of 5184 × 3888 pixels. The images were collected during May and June of the 2021 and 2022 growing seasons from commercial tomato fields in Santa Amalia, Badajoz, an important agricultural hub in Spain’s Vegas Altas region, known for its intensive tomato cultivation and processing facilities. The dataset contains 1217 JPG images and 21,208 labelled instances. It is divided into two subsets: the first includes 938 images and 9060 instances from 2021, while the second comprises 278 images and 11,931 instances from 2022. Each image is manually annotated to identify individual plant species. This dataset is intended for training and evaluating advanced deep learning models, including convolutional neural networks and vision transformers, to enable early-stage weed detection and classification. By making it publicly accessible, the study supports the development of image-based monitoring systems that improve the efficiency, accuracy, and environmental sustainability of precision agriculture practices.

Specifications Table

**Table:** 

Subject	Computer Sciences
Specific subject area	Computer Vision and Pattern Recognition. Agronomy and Crop Science.
Type of data	Raw
Data collection	The dataset comprises RGB images captured during the early growth stages of commercial tomato crops naturally infested with weeds. Image acquisition was carried out under clear, sunny conditions during midday hours in May and June of the 2021 and 2022 growing seasons. The data are organized into two subsets, corresponding to each respective year. Each image was manually annotated by experts in weed science using the labelImg software, following a visual identification of plant species. The annotations adhere to the PASCAL VOC, YOLO, and COCO JSON format. The dataset encompasses five agriculturally relevant weed species, categorized into monocotyledons (*Cyperus rotundus* L., *Echinochloa crus-galli* L., *Setaria verticillata* L.) and dicotyledons (*Portulaca oleracea* L., *Solanum nigrum* L.). The cultivated tomato (*Solanum lycopersicum* L.) is also represented, particularly within the 2021 subset.
Data source location	Data collected from commercial tomato fields in Santa Amalia, Badajoz, Spain. Datum ETRS89. Coordinates: 38°59′46.36″ N, 6°03′48.39″ W. UTM Zone 29: *X* = 754,318.63 m, *Y* = 4320,459.16 m
Data accessibility	Repository name: GROUNDBASED_WEED: Ground-Based Imagery for Early Weed Classification in Tomato Crops [Data set]; DIGITAL.CSIC Data identification number: https://doi.org/10.20350/digitalCSIC/17475 Direct URL to data: https://doi.org/10.20350/digitalCSIC/17475
Related research article	Adrià Gómez, Hugo Moreno, Constantino Valero, Dionisio Andújar, Spatio-temporal stability of intelligent modeling for weed detection in tomato fields, Agricultural Systems, Volume 228, 2025, 104,394, ISSN 0308–521X, https://doi.org/10.1016/j.agsy.2025.104394.

## 1 Value of the Data

• GROUNDBASED_WEED comprises a comprehensive collection of images depicting tomato crop and weeds within highly complex and occluded backgrounds, establishing a critical resource for researchers engaged in the development of site-specific weed management.
• GROUNDBASED_WEED addresses the scarcity of open-access, annotated imagery of early-stage weeds in tomato and maize rotations, a prerequisite for training scalable deep learning architectures and implementing precision weed control strategies.
• GROUNDBASED_WEED includes labels for both tomato crop and weeds, supporting not only weed detection but also crop identification. These systems can leverage the identified crop regions to infer the presence of weeds not explicitly represented in this dataset.
• GROUNDBASED_WEED enables weed species identification, feature extraction, and the training and evaluation of deep learning models, such as Convolutional Neural Networks and Vision Transformers. By providing high-resolution, ground-based RGB imagery annotated at the instance level, the dataset enables the development and training of deep learning models for accurate weed identification at early phenological stages.
• GROUNDBASED_WEED supports the development of autonomous, site-specific herbicide application systems and advances computer vision techniques for object detection and classification.
• GROUNDBASED_WEED dataset supports multiclass classification and artificial image generation for data augmentation.

## 2 Background

The GROUNDBASED_WEED dataset contains images annotated in JPG format, with each instance manually labelled by weed science experts following rigorous visual identification protocols. The annotation process adheres to standardized formats, enabling interoperability with common deep learning frameworks. Tomatoes are globally significant, ranking as the second most-produced vegetable crop by quantity after potatoes [1]. Furthermore, the dataset incorporates weed species typically associated with rotational systems involving maize, a major cereal frequently integrated into tomato-based cropping sequences. Thus, this dataset was developed to mitigate the current scarcity of open-access, annotated imagery focused on the early phenological stages of weed species within tomato crop environments. Such data are essential for training and validating scalable deep learning architectures, including Convolutional Neural Networks (CNNs) and Vision Transformers (ViTs), which underpin advanced weed detection and classification systems. Moreover, deep learning models require high-quality, diverse, and large-scale data for robust performance [2], highlighting the need for open-access datasets [3]. Therefore, by providing standardized, high-resolution images with expert annotations, this dataset facilitates the development of robust, generalizable models for precision weed management, enabling both chemical and mechanical control strategies within site-specific agriculture frameworks. In addition, it can be employed to train generative artificial intelligence models aimed at synthesizing realistic artificial imagery [4]. Thus, enabling dataset augmentation and reducing the dependence on labor-intensive manual annotation [5].

## 3 Data Description

This dataset comprises 1216 high-resolution RGB images (5184 × 3888 pixels) captured under natural lighting conditions in commercial tomato fields located in Santa Amalia, Badajoz, Spain. The images were acquired during the early growth stages of both tomato plants and associated weed species, coinciding with the typical weed treatment period. Importantly, no herbicide applications were carried out during image collection or in the preceding 10 days. Data were collected during the 2021 and 2022 growing seasons (May–June), representing typical summer cropping conditions in the Vegas Altas agricultural region. The images are grouped into two folders based on the year of collection: TOMATO_1 (2021), containing 938 images with 9060 annotated instances, and TOMATO_2 (2022), comprising 278 images with 11,931 annotated instances. To ensure optimal object visibility, a zenithal perspective was maintained at an approximate height of 1 to 1.5 m above the canopy. Seven plant classes were annotated using the EPPO code system, including five prevalent weed species—*Cyperus rotundus* L. (CYPRO), *Echinochloa crus-galli* L. (ECHCG), *Setaria verticillata* L. (SETVE), *Portulaca oleracea* L. (POROL), and *Solanum nigrum* L. (SOLNI)—as well as the crop species *Solanum lycopersicum* L. (LYPES), and a non-recognized (NR) category for unclassified individuals (Table 1).

**Table 1:** Summary of annotated crop and weed species and their relative frequencies in the dataset.

Scientific name	EPPO Label	Instancesᵃ	Percentage
*Solanum lycopersicum* L.	LYPES	1075	5.12 %
*Solanum nigrum* L.	SOLNI	6322	30.12 %
*Portulaca oleracea* L.	POROL	1761	8.39 %
*Cyperus rotundus* L.	CYPRO	3787	18.04 %
*Setaria verticillata* L.	SETVE	1790	8.53 %
*Echinochloa crus-galli* L.	ECHCG	5322	25.35 %
Not recognized	NR	934	4.45 %
Total Instances		20,991	100.00 %

ᵃ The annotated instances are distributed across 1216 images collected over a two-year sampling period.

**Fig. 1:**
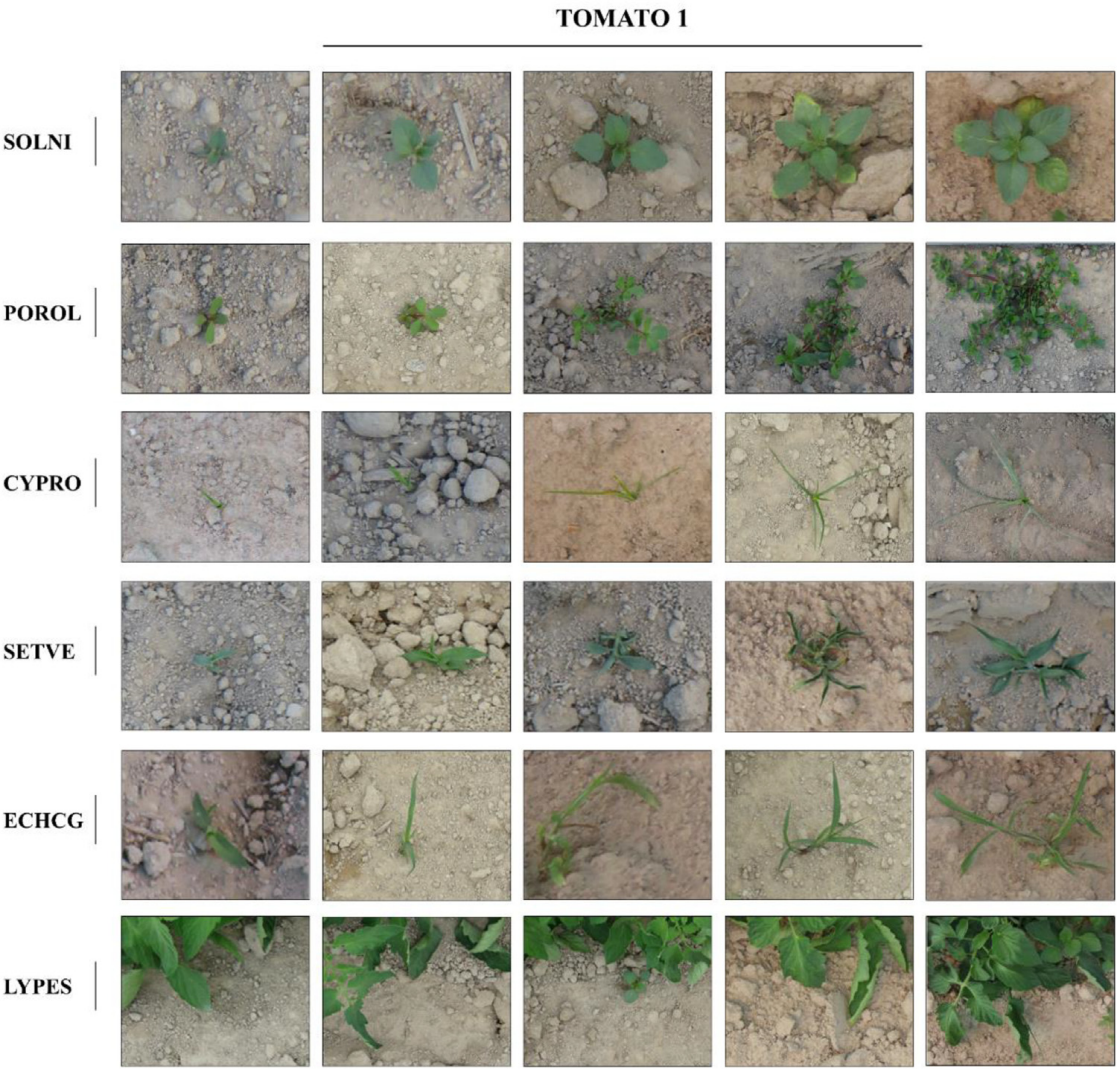
Examples of labelled images in TOMATO_1. The weed species are: SOLNI for plants identified as the species *Solanum nigrum* L., POROL for plants identified as the species *Portulaca oleracea* L., SETVE for plants identified as the species *Setaria verticillata* L., ECHCG for plants identified as the species *Echinochloa crus galli* L, CYPRO for plants identified as the species *Cyperus rotundus* L. LYPES for the crop identified as the species *Solanum lycopersicum* L.

**Fig. 2:**
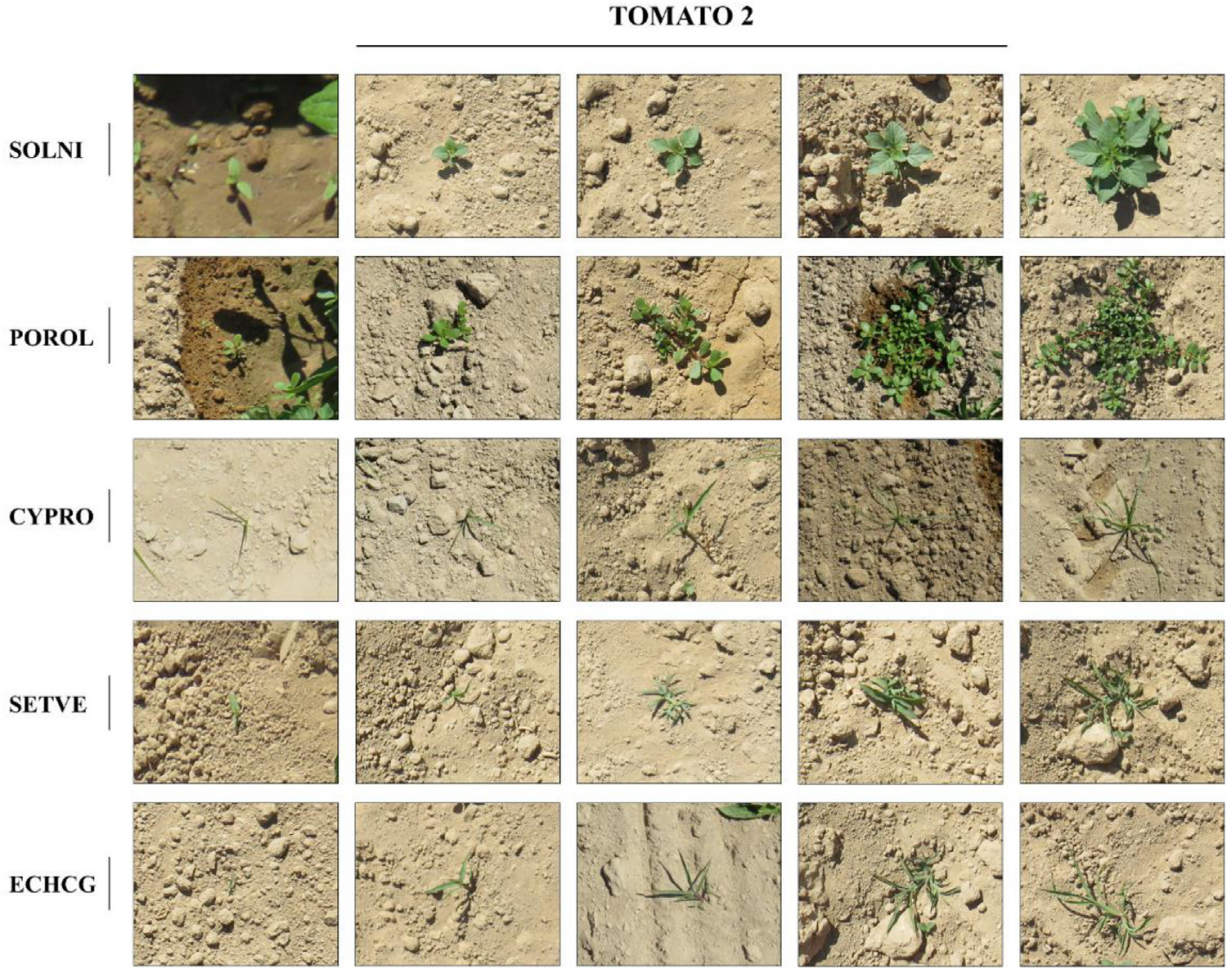
Examples of labelled images in TOMATO_2. The weed species are: SOLNI for plants identified as the species *Solanum nigrum* L., POROL for plants identified as the species *Portulaca oleracea* L., SETVE for plants identified as the species *Setaria verticillata* L., ECHCG for plants identified as the species *Echinochloa crus galli* L, CYPRO for plants identified as the species *Cyperus rotundus* L.

Annotations were conducted using LabelImg (v1.8.6) in PascalVOC XML, YOLO, and COCO JSON format, including bounding box coordinates and class labels. On the other hand, this dataset was collected to support research on weed classification and to facilitate the development of deep learning models for weed detection, spatio-temporal analysis, and the generation of artificial weed imagery [4,5]. Owing to its broad range of annotated categories and substantial volume of high-resolution images, the dataset provides a valuable resource for training and evaluating computer vision algorithms capable of accurately distinguishing between weed and crop species. The annotated images are provided in JPG format with varying spatial dimensions, covering the full visible area of the specimens. File names indicate the annotated species (weed or crop). Fig. 1, Fig. 2 present representative examples of early-stage weed and crop instances captured in the dataset for TOMATO_1 and TOMATO_2 respectively.

**Fig. 3:**
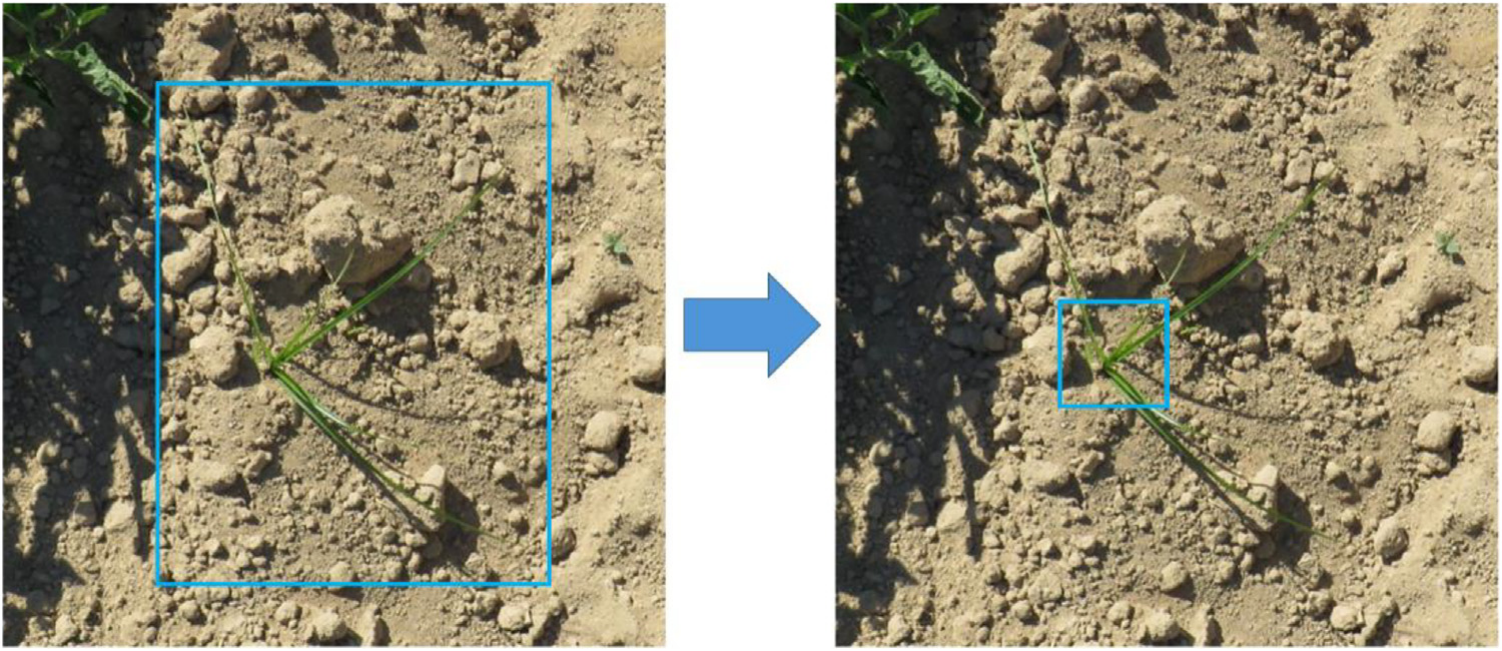
The image depicts the labelling inconsistency observed in the dataset. To ensure uniformity, all CYPRO instances were reannotated according to a standardized labelling protocol.

Unlike the other annotated species in the dataset, *Cyperus rotundus* L. (CYPRO) required a different labeling strategy to ensure accurate detection. Upon further investigation in [2] and in also agreement with Correa*,* et al. [6] it was found that this issue stemmed from inconsistencies in annotation across the two years: in the 2021 training dataset, CYPRO instances were labelled by outlining the entire plant, whereas in the 2022 testing dataset, only the central portion of the weed was annotated. Fig. 3 highlights this labelling discrepancy. To address this, all CYPRO instances were reannotated to follow a consistent labelling protocol.

## 4 Experimental Design, Materials and Methods

### 4.1 Field data collection

**Fig. 4:**
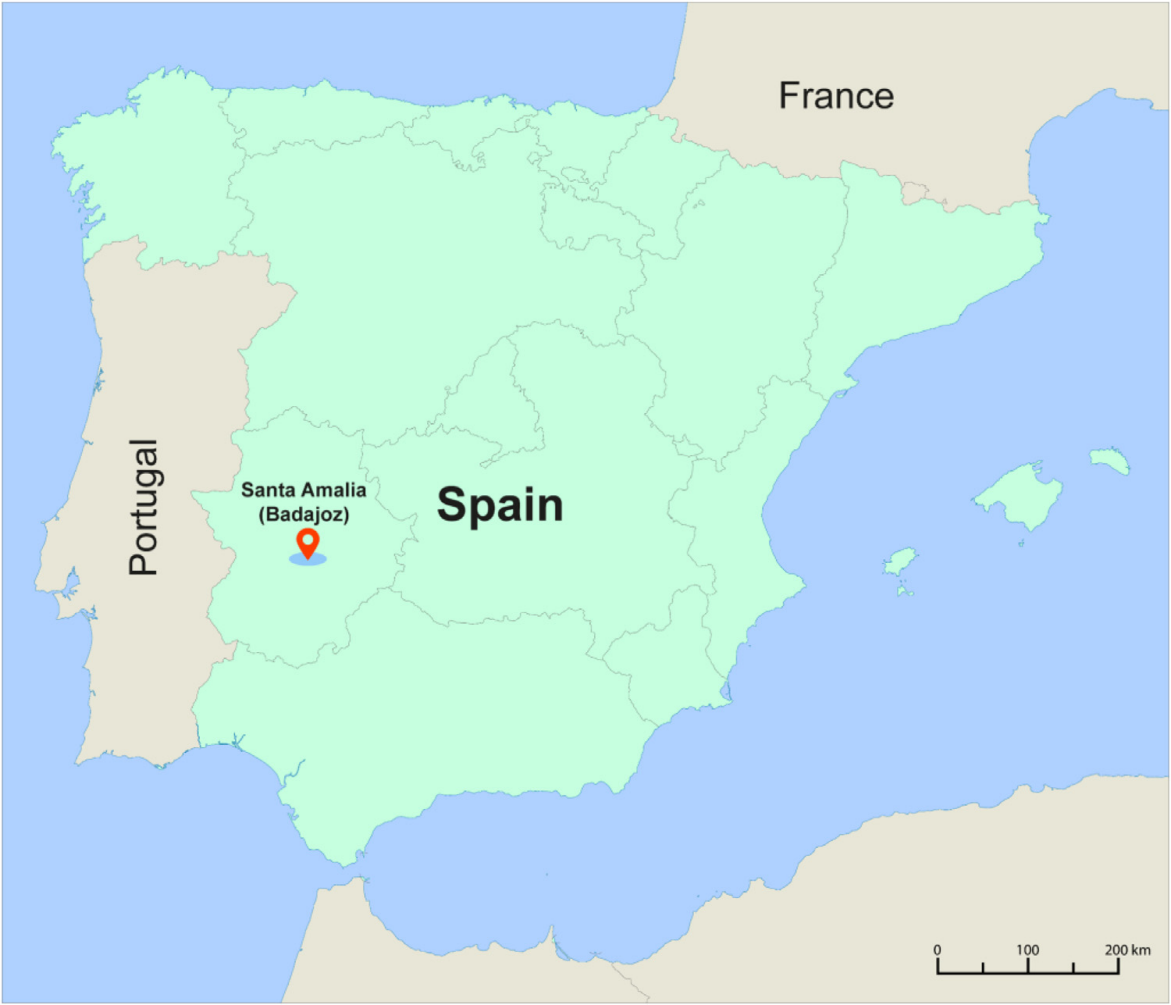
Geographic location of the sampling site in Spain. The tomato crop fields surveyed in the study are located in commercial agricultural areas in Santa Amalia, Badajoz, and are indicated by the red marker.

The images were acquired under natural lighting conditions in open-field commercial tomato plantations located in Santa Amalia, Badajoz (38°59′46.36″ N, 6°03′48.39″ W; datum: ETRS89). In UTM Zone 29, this location corresponds to coordinates *X* = 754,318.63 m and *Y* = 4320,459.16 m (Fig. 4). The dataset was collected across five distinct fields covering a total area of 36 ha. This site lies within the Vegas Altas region of southwestern Spain, a prominent agricultural area characterized by intensive horticultural production [7].

Image acquisition was conducted manually using a Canon PowerShot SX540 HS camera, which provides a spatial resolution of 5184 × 3888 pixels. To ensure optimal image sharpness and reduce motion blur, a shutter speed of 1/1000 was employed, while the ISO was automatically adjusted to accommodate variations in ambient light. Table 2 presents a summary of camera specifications and technical details of image acquisition. The images were captured at an approximate ground sampling distance (GSD) of 0.05 cm pixel⁻¹, allowing for the detailed visualization of plant structures at early growth stages. This acquisition protocol was designed to generate high-quality visual data suitable for advanced image analysis and the development of computer vision algorithms for weed and crop identification.

**Table 2:** Camera specifications and image acquisition settings.

Specification	Details
Camera Model	Canon PowerShot SX540 HS
Sensor Type	20.3 MP CMOS
Lens	Built-in 50 × optical zoom lens
Focal Length	4.3–215.0 mm (35 mm equivalent: 24–1200 mm)
Aperture Range	f/3.4 (wide) to f/6.5 (telephoto)
Image Resolution	5184 × 3888 pixels
ISO Sensitivity	AUTO (ISO range: 80–1600)
Shutter Speed Range	1/1000 s (default); 15–1/2000 s (full range)
Shooting Mode Used	Program AE (for ISO configuration)
Perspective	Zenithal (1–1.5 m above ground)
Lighting Conditions	Natural daylight, no flash used
Image Format	JPG

### 4.2 Species labeling

**Fig. 5:**
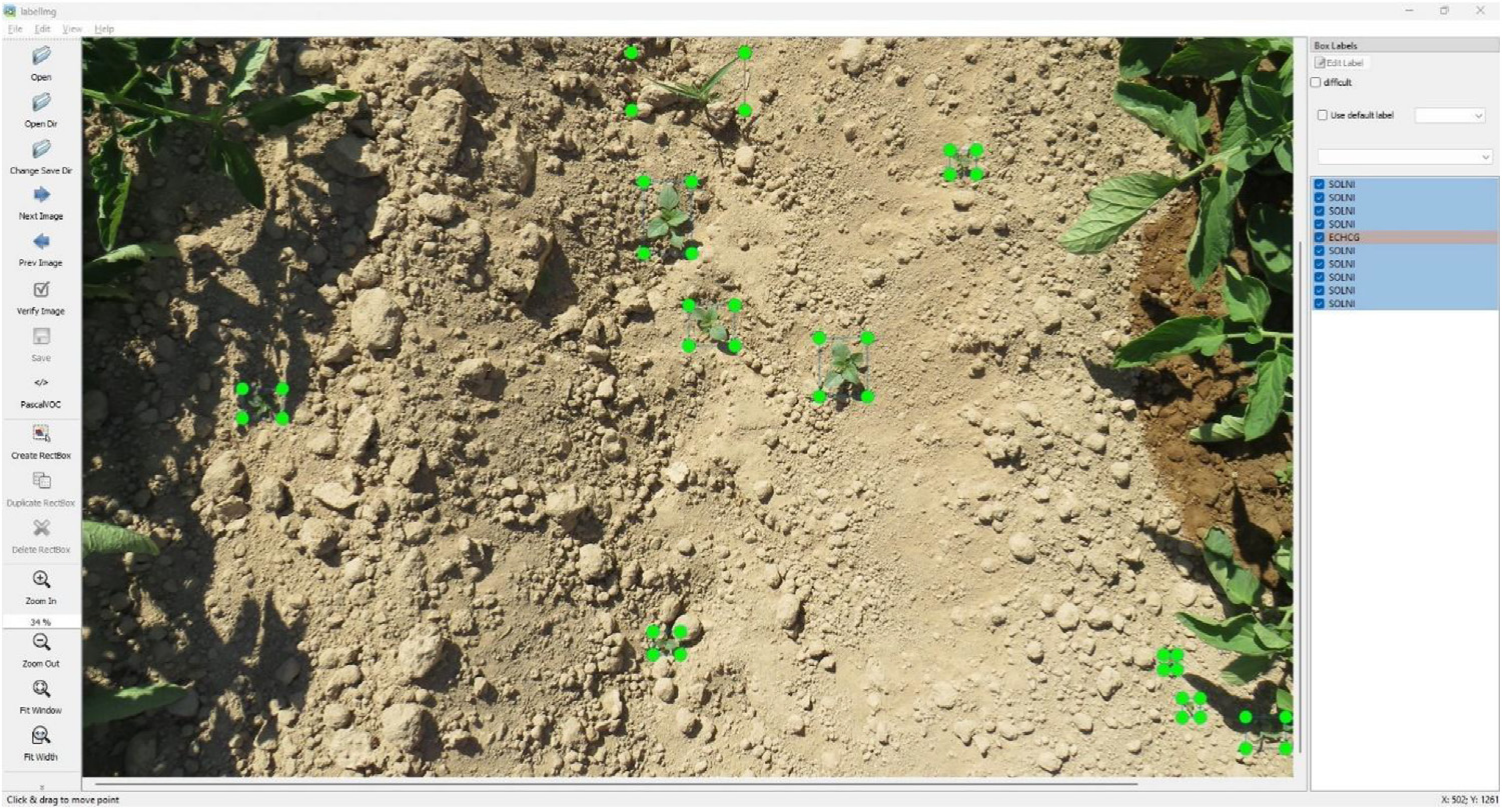
Example of a ground-level view of a tomato crop field showing weed plants growing between the rows. The image is displayed within the LabelImg annotation tool interface, where individual weed species are annotated using bounding boxes. The left panel of the interface provides navigation and annotation tools, while the right panel displays the list of label categories, each accompanied by a selectable checkbox.

To ensure consistency and reliability in the annotation process, a series of quality assurance measures was implemented during the development of the GROUNDBASED_WEED dataset. Prior to image acquisition, a preliminary field survey was conducted by two weed science specialists to systematically identify and document the weed species present in tomato crops. This preparatory step provided a robust foundation for the subsequent annotation phase. The same experts performed image annotation to minimize individual subjectivity and enhance consistency in species identification across the dataset. Annotators visually inspected each image, drew bounding boxes around clearly visible and fully intact plants, and assigned the appropriate species label. Plants that were truncated by image borders or insufficiently visible were excluded from labeling to prevent potential misclassification. Each image was manually labeled using the LabelImg tool (version 1.8.6) (Fig. 5) on a Windows 10 (64-bit) operating system. Moreover, the EPPO coding system was applied to maintain uniformity throughout the process. Instances where plant identification remained uncertain (due to limited visibility, image resolution, or partial representation) were intentionally left unannotated. This approach ensured that only high-confidence annotations were retained, thereby preserving the integrity of the dataset. To further guarantee quality, consistency checks were conducted to identify and resolve discrepancies. For example, variations in annotation strategies for species such as *Cyperus rotundus* L. (CYPRO) across different years were addressed through reannotation using a standardized approach (Fig. 3). Collectively, these measures ensured a high level of annotation accuracy and reliability, making the dataset well-suited for training robust deep learning models.

**Fig. 6:**
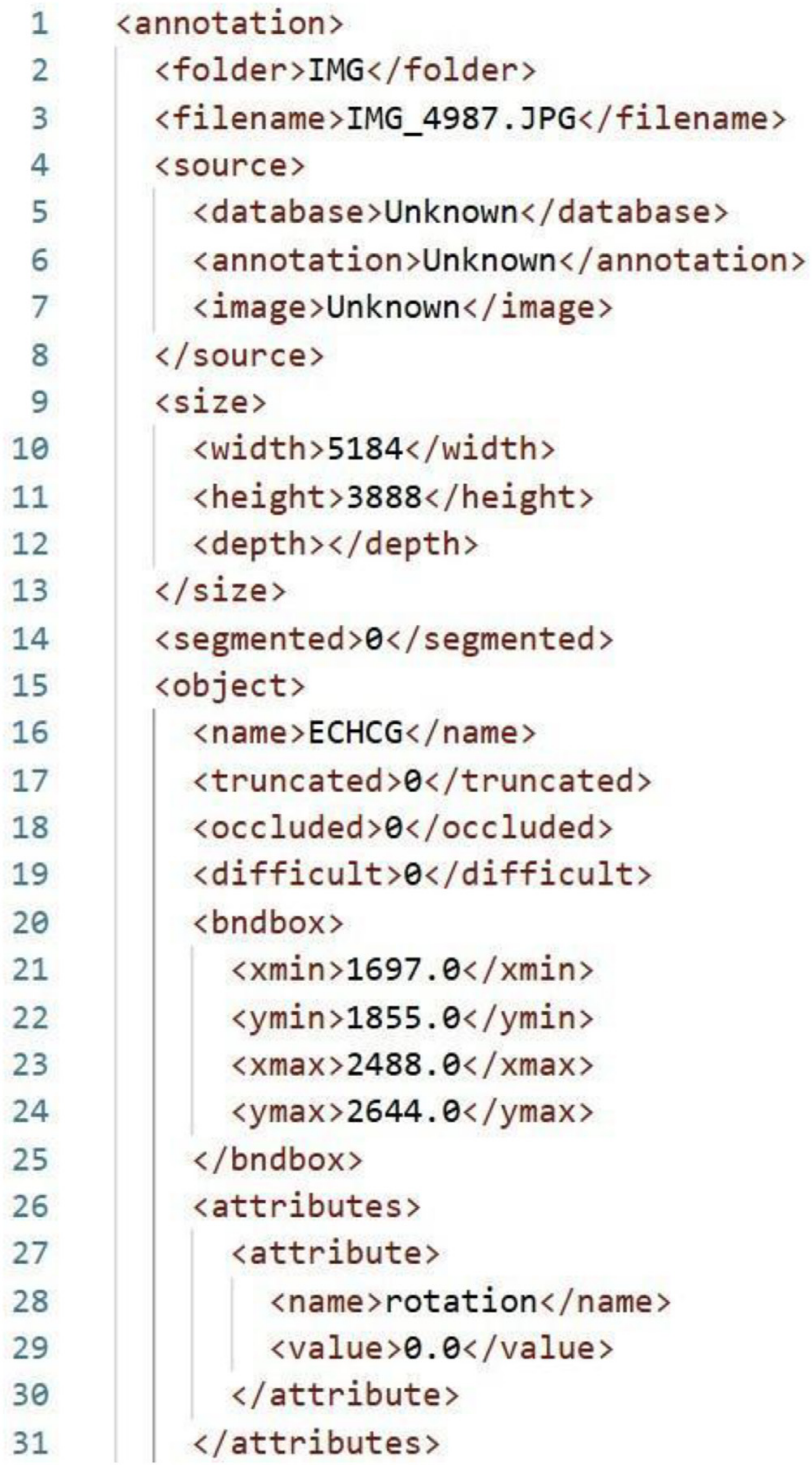
Example of a file generated during labelling, following the PASCAL VOC convention format. The XML annotation corresponds to the image IMG_4987.JPG and includes metadata such as image dimensions and object details. This format supports structured data for object detection tasks.

The annotations were exported in Pascal VOC XML format (Fig. 6), which records the spatial coordinates of each bounding box—specifically the top-left (xmin, ymin) and bottom-right (xmax, ymax) corners—alongside the corresponding class label. Each annotated image was associated with a single XML file containing all identified objects within that image. YOLO and COCO JSON format is also provided. Seven plant classes were labelled with the assistance of weed experts, following the standardized coding system of the European and Mediterranean Plant Protection Organization (EPPO). These included the weed species of *Solanum nigrum* L. (SOLNI), *Portulaca oleracea* L. (POROL), *Setaria verticillata* L. (SETVE), *Echinochloa crus-galli* L. (ECHCG), *Cyperus rotundus* L. (CYPRO), and *Solanum lycopersicum* L. (LYPES) as the crop, the latter representing the crop species. An additional class, labelled as NR (Not Recognized), was used for plants that could not be reliably identified due to insufficient image clarity or ambiguous features. The resulting annotation protocol, grounded in expert validation, provides a robust foundation for developing and benchmarking advanced computer vision models aimed at the early detection and classification of weed and crop species.

### 4.3 Organization and storage of the dataset

To improve accessibility and support targeted research in computer vision and precision agriculture, the dataset was methodically structured to ensure efficient handling, navigation, and reuse. The data were collected during the 2021 and 2022 growing seasons, with field acquisition campaigns conducted in May and June of each year to capture the early phenological stages of tomato crops and their associated weed species. To preserve the temporal integrity of the data, the dataset is organized into two main directories: TOMATO_1, containing 938 high-resolution images and 9060 annotated instances from the 2021 season, and TOMATO_2, comprising 278 images and 11,931 labelled instances from the 2022 season, as summarized in Table 3. This temporal separation enables researchers to assess model robustness and generalization across growing seasons and different stages of plant development. Within each temporal folder—TOMATO_1 and TOMATO_2—the data are further divided into two subfolders: images, which contain the original RGB images in JPG format, and annotations, which include the corresponding XML files structured according to the Pascal VOC standard. These annotation files provide essential metadata for supervised learning tasks, including the bounding box coordinates and the class labels for each annotated object. The dataset was compiled using a semi-automated workflow to ensure consistency, reproducibility, and scalability. This process involved extracting the bounding box information from the XML annotation files, cropping individual weed instances from the subdivided image tiles, assigning a unique identifier to each instance based on its original location, and categorizing each object according to its species label.

**Table 3:** Species-wise Annotation Statistics and Temporal Breakdown of the GROUNDBASED_WEED Dataset. Labelling weed species by EPPO code (European and Mediterranean Plant Protection Organization).

Scientific name	Label	TOMATO_1	TOMATO_2	Total instances	%
		Instances			
*Solanum lycopersicum* L.	LYPES	1075	0	1075	5.12 %
*Solanum nigrum* L.	SOLNI	2657	3665	6322	30.12 %
*Portulaca oleracea* L.	POROL	657	1104	1761	8.39 %
*Cyperus rotundus* L.	CYPRO	2486	1301	3787	18.04 %
*Setaria verticillata* L.	SETVE	1255	535	1790	8.53 %
*Echinochloa crus-galli* L.	ECHCG	238	5084	5322	25.35 %
Not recognized	NR	692	242	934	4.45 %
		9060	11,931	20,991	100 %

## Limitations

Each crop and weed species have specific ecological requirements for optimal growth. However, the environmental conditions during the data-collection period—such as temperature, water availability, soil characteristics, and other abiotic factors—may not have been uniformly favorable for all species. Hence, the dataset is not balanced. In TOMATO 1, the dataset is dominated by Solanum nigrum and *Cyperus rotundus* L., which together account for more than half of the labels. In contrast, *Echinochloa crus-galli* L. is extremely scarce, representing only 2.6 % of the labels. A model trained mainly on these images will therefore have little opportunity to learn the appearance of *Echinochloa crus-galli* L, increasing the likelihood of false negatives for that weed. In TOMATO 2, the situation changes: *Echinochloa crus-galli* L becomes the single largest class, comprising 42.6 % of the labels. As a result, the dataset may not fully represent the diversity of weed flora that could affect crop yield and quality in future sampling efforts. For example, weed species such as *Chenopodium album* L., *Amaranthus retroflexus* L., and *Amaranthus palmeri* L., which are commonly associated with tomato cultivation and exert significant competitive pressure on crop development, may appear in future sampling collections.

## Ethics Statement

The authors have read and follow the ethical requirements for publication in Data in Brief and confirm that the current work does not involve human subjects, animal experiments, or any data collected from social media platforms.

## Credit Author Statement

**Hugo Moreno:** Conceptualization, Methodology, Investigation, Data curation, Visualization, Writing – original draft; **Gabriel Rivera:** Data curation, Visualization and Writing – original draft; **Dionisio Andújar:** Conceptualization, Methodology, Investigation, Resources, Writing - Review & Editing, Supervision, Project administration, Funding acquisition.

## Data Availability

XXXXGROUNDBASED_WEED: Ground-Based Imagery for Early Weed Classification in Tomato Crops (Original data).
